# Shikonin promotes hypertrophic scar repair by autophagy of hypertrophic scar-derived fibroblasts

**DOI:** 10.1590/acb384623

**Published:** 2023-10-23

**Authors:** Qing Zhang, Maomao Wang, Xingwang Deng, Dan Zhao, Fang Zhao, Jinli Xiao, Jiaxiang Ma, Xiaoliang Pan

**Affiliations:** 1 Ningxia Medical University General Hospital – Tissue Organ Bank & Tissue Engineering Centre – c (Ningxia) – China.; 2 Ningxia Medical University – Key Laboratory of Fertility Preservation and Maintenance of Ministry of Education – School of Basic Medicine – Yinchuan (Ningxia) – China.; 3 Ningxia Medical University – Clinical Medical School – Yinchuan (Ningxia) – China; 4 The First People’s Hospital – Department of Burns and Plastic Surgery – Shizuishan – China.

**Keywords:** Shikonin, Autophagy, Cicatrix

## Abstract

**Purpose:**

To investigate the Shikonin (SHI) induce autophagy of hypertrophic scar-derived fibroblasts (HSFs) and the mechanism of which in repairing hypertrophic scar.

**Methods:**

This study showed that SHI induced autophagy from HSFs and repaired skin scars through the AMPK/mTOR pathway. Alamar Blue and Sirius red were used to identify cell activity and collagen. Electron microscopy, label-free quantitative proteomic analysis, fluorescence and other methods were used to identify autophagy. The differences in the expression of autophagy and AMPK/mTOR pathway-related proteins after SHI treatment were quantitatively analyzed by Western blots. A quantitative real-time polymerase chain reaction assay was used to detect the expression of LC3, AMPK and ULK after adding chloroquine (CQ) autophagy inhibitor.

**Results:**

After treatment with SHI for 24 hours, it was found that the viability of HSFs was significantly reduced, the protein expression of LC3-II/LC3-I and Beclin1 increased, while the protein expression of P62 decreased. The expression of phosphorylated AMPK increased and expression of phosphorylated mTOR decreased. After the use of CQ, the cell autophagy caused by SHI was blocked. The key genes LC3 and P62 were then reexamined by immunohistochemistry using a porcine full-thickness burn hypertrophic scar model, and the results verified that SHI could induce autophagy *in vivo*.

**Conclusions:**

These findings suggested that SHI promoted autophagy of HSFs cells, and the potential mechanism may be related to the AMPK/mTOR signal pathway, which provided new insights for the treatment of hypertrophic scars.

## Introduction

In one-three months after deep skin trauma, such as physical injury, burns or surgery, a wound can develop an excessive healing reaction, which will release pro-fibrotic factors and inflammatory factors, resulting in excessive proliferation of fibroblasts and excessive deposition and degradation of extracellular matrix (ECM), inducing a hypertrophic scar (HS)^1^, which may be red or purplish red, irregular in shape, protruding on the skin surface and often accompanied by varying degrees of itching and nerve pain^2^. Severe HS not only affects the appearance of patients, but causes dysfunction, which impacts detrimentally on the patients’ lives^3^.

**Figure 1:**
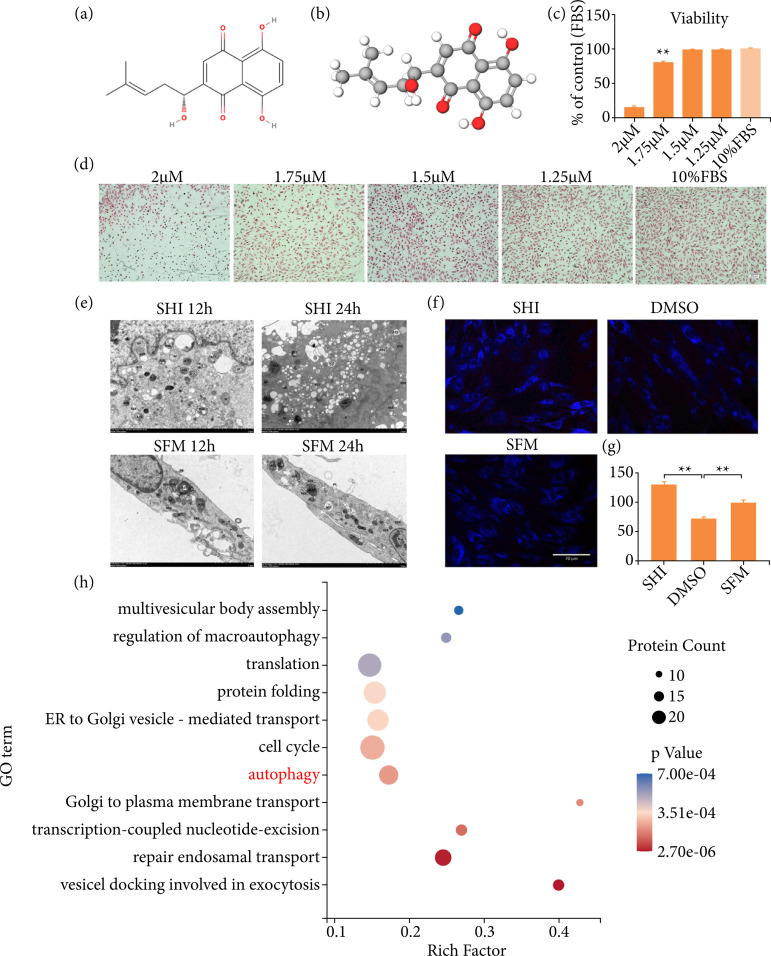
Inhibitory effects of SHI on HSFs. **(a)** Two-dimensional chemical formula visualization of SHI (C_16_H_16_O_5_; 288.295 Da). **(b)** Three-dimensional chemical formula visualization of SHI. **(c)** Quantification of HSF viability under different concentration of SHI for 24 hours. **(d)** Sirius red staining demonstrated SHI decreased collagen deposition in HSFs compared with control group. **(e)** TEM images of HSFs induced by SHI at 12 and 24 hours (×7,000). **(f)** Representative images of fluorescence microphotographs and the fluorescent intensity of autophagosomes of different groups. scale bar = 70 μM. **(g)** Quantification of autophagosomes of different groups. **(h)** GO enrichment analysis: autophagy-related proteins were highly expressed in HSFs treated with SHI. Chemical structure obtained from MolView website (https://molview.org/).

SHI: Shikonin; DMSO: methyl sulphoxide; SFM: serum free medium; HSF: hypertrophic scar-derived fibroblasts; GO: gene ontology; PS: pseudopodium; N: nucleus; Nu: nucleolus; M: mitochondria; RER: rough endoplasmic reticulum; AP: autophagosome; ASS: autolysosome; n = 3; *significant difference with P < 0.01; **significant difference with P < 0.05. Source: Elaborated by the authors.

The treatment of HS depends on the body region and severity of scar contracture^4^. At present, available treatment modalities include oral medicines, the application of topical and intralesional steroids, rest / fixation therapy, compression therapy, mesenchymal stem cell therapy, cryotherapy, laser therapy, radiation, fat grafting, surgical excision and so on, each with varying degrees of success^1^,^4^,^5^. The treatment of HS caused by skin fibrosis is a complex process, and the pathogenesis is not completely clear^6^. So far, no drug has been approved as a complete inhibitor of fibrosis^7^, but some drugs such as Shikonin (SHI) have been used in the treatment of skin fibrosis. It is a naphthoquinone isolated from traditional medicinal plant species seen in Figs. 1a and 1b and has been used historically to treat measles, sore throats and burns, due to its anti-inflammatory, anti-gonadotropin, and anti-tumor properties^8^,^9^. The data of several studies have provided evidence to support the potential use of SHI as a new therapy for hypertrophic scarring^10^,^11^.

Autophagy is a highly conserved cellular process, which plays an important role in many physiological and disease processes and is induced by various stressors such as hunger and hypoxia^12^. Studies have shown that, compared with normal skin tissue, the levels of Light Chain 3 (LC3), Beclin1 and proteins in scar tissue were down-regulated, and the reduced autophagy ability may be related to the pathogenesis of HS^13^. So, autophagy is closely related to the formation and development of scars. Studies have shown that SHI induced autophagy in clinical conditions such as BXPC-3 human pancreatic cancer cells^14^ and human melanoma A375 cells^15^, but SHI-induced autophagy in scar fibroblasts has not been reported.

In this study, the effects of different concentrations of SHI on the activity of human proliferative scar fibroblasts and the changes of autophagosome activity were examined, and the regulatory effect and molecular mechanism of SHI-induced autophagy on the expression of human proliferative scar fibroblasts were further analyzed, to create a new theoretical foundation for the treatment of human proliferative scars using SHI.

## Methods

### Cell culture

Hypertrophic scar-derived fibroblasts (HSFs) were isolated from three different participants (etiology: burns; age: 20 to 44 years old) with HS presented at least six months and had received a cicatrectomy in the Department of Burn Surgery of General Hospital of Ningxia Medical University (Yinchuan, China), and informed consent was provided. Briefly, the collected tissue samples were washed by phosphate buffer solution (PBS) for 30 minutes at room temperature and cut into small pieces. Then, they were digested with 0.05% trypsin (Gibco, Grand Island, NY, United States of America) and 0.1% collagenase type I (Gibco, Grand Island, NY, United States of America) at 37°C for 2 hours separately. The cells from digested tissue samples were cultured by Dulbecco’s modified eagle’s medium (DMEM, Gibco, Grand Island, NY, United States of America) with 10% fetal bovine serum (FBS, Gibco, Grand Island, NY, United States of America) medium under 5% CO_2_ and 37°C. The cultured cells were used between the fourth and seventh passages for further research^16^.

### Sirius red staining

Extract SHI powder was produced by the National Institute for the Control of Pharmaceutical and Biological Products, Beijing, China. Sirius red (SR) staining was performed to analyze the collagen in the cells. Cells were inoculated in a 48-well plate, cultured with 10% FBS medium for 24 hours and then added to 2, 1.75, 1.5 and 1.25 μM SHI. After 24 hours, 0.1% v/v SR (Solarbio, Beijing, China) was added into wells for 90 minutes, and images were then taken by CKX53 inverted microscope (Olympus, Tokyo, Japan) with camera^17^.

### Cell viability assay

The cell viability was examined with Alamar blue (AB) (Sigma-Aldrich, St. Louis, MO, United States) assay to observe the viability of HSFs caused by SHI. The changes in HSFs viability were then quantified, and florescence was determined at λex 560 nm and λem 590 nm in an Infinite 200 Pro Microplate Reader (Tecan, Mannedorf, Switzerland)^16^. The percent viability was calculated using the Eq. 1:


Cell viability(%)=treated/control×100% (1)

### Transmission electron microscope

Cells were treated with 1.75 μM SHI for 12 or 24 hours and then collected in a serum free medium (SFM) as the control group. Cells were fixed with 2.5% glutaraldehyde (Solarbio, Beijing, China) for 2 hours and treated with 1% osmium tetroxide (Sigma-Aldrich, St. Louis, MO, United States of America) for 2 hours. Then, the sample was embedded with epoxy resin (Sigma-Aldrich, St. Louis, MO, United States of America) after dehydrated through a graded series of ethanol and polymerized at 60°C for 48 hours. Samples were cut into ultrathin sections of 60 to 80 nm by an EM UC7 ultramicrotome (Leica, Solms, Germany) and stained with 2% uranyl acetate and lead citrate. The images were acquired using an HT7700 TEM (Hitachi, Tokyo, Japan).

### LC-MS/MS label-free quantitative proteomics and bioinformatics analyses

The HSFs treated by SHI for 24 hours and untreated HSFs were collected to prepare the whole-cell lysates. A bicinchoninic acid (BCA) kit (ThermoFisher, Wilmington, DE, United States of America) was used to measure protein concentration. Dithiothreitol (Sigma-Aldrich, St. Louis, MO, United States of America) and iodoacetamide (Sigma-Aldrich, St. Louis, MO, United States of America) were added into the protein solution and incubated at room temperature in dark for 15 minutes, then incubated overnight with trypsin. The tryptic peptides were fractionated by high pH reverse-phase high-performance liquid chromatography (HPLC) using a 300Extend C18 column of 5 μM particles, 4.6 mm ID, 250 mm length (Agilent, Santa Clara, CA, United States of America). The tryptic peptides were dissolved in 0.1% formic acid (solvent A, Fluka, Buchs, Switzerland) and separated using 0.1% formic acid in 98% acetonitrile (solvent B, ThermoFisher, Wilmington, DE, United States of America). The created gradient was 6 to 22% solvent B for 0 to 40 minutes, 22 to 35% solvent B for 40 to 52 minutes, 35 to 80% solvent B for 52 to 56 minutes and 80% solvent B for 56 to 60 minutes at a flow rate of 700 nL/minute on an EASY-nLC 1000 UPLC system (ThermoFisher, Wilmington, DE, United States of America).

The mass spectrometry analysis was performed on Orbitrap FusionTM lumos (ThermoFisher, Wilmington, DE, United States of America). The scanning range of primary MS was 350 to 1,550 m/z, and the scanning resolution was 60,000. The secondary scan resolution was 15,000, and data was retrieved by Maxquant search engine (v 1.5.2.8). The differential protein information was uploaded to the UniProt-GOA database (https://www.ebi.ac.uk/GOA) to enrich and analyze the gene ontology (GO) function of the differentially expressed protein.

### Autophagy assay for hypertrophic scar-derived fibroblasts

Each well of 96-well plates were seeded with 10,000 to 20,000 cells in 10% FBS for 24 hours, then treated with 1.75 μM SHI dissolved with methyl sulphoxide (DMSO, Sigma-Aldrich, St. Louis, MO, United States of America) for 24 hours, DMSO and SFM as the control group. The data of cells were collected from three patients, and the experiment was repeated three times. A MAK138-1KT autophagy assay kit (Sigma-Aldrich, St. Louis, MO, United States of America) was used to measure the autophagosomes of the HSFs.

Cells were imaged by a microscope system (Echo Laboratories, San Diego, CA, United States of America) with a DAPI channel, and the fluorescence absorbance at 360 and 520 nm was measured using a Tecan Austria GmbH Infinite M200 PRO plate reader.

Image J software (NIH, Bethesda, MD, United States of America) was used to identify and quantify autophagosomes in cells automatically and compare autophagy levels between different groups by GraphPad Prism version 7 (GraphPad Software, Boston, MA, United States of America). Statistical analysis used one-way analysis of variance (ANOVA) with the Student’s t-test.

### Western blot analysis

The cells were lysed by a protein extraction kit (KeyGEN, Nanjing, China). The concentrations of protein were measured by BCA kit. The proteins were separated by SDS gel electrophoresis and transferred to a polyvinylidene difuoride membrane for 2.5 hours. The membrane was blocked with 5% skim milk for 1 hour at room temperature. The membrane was incubated at 4°C overnight with rabbit monoclonal anti-human LC3 (Santa Cruz Biotechnology, Dallas, TX, United States of America), P62 (Cell Signaling Technology, Beverly, MA, USA), Beclin1 (Proteintech, Manchester, United Kingdom), AMP-activated protein kinase (AMPK) (Cell Signaling Technology, Beverly, MA, United States of America), p-AMPK (Cell Signaling Technology, Beverly, MA, USA), mammalian target of rapamycin (mTOR) (Santa Cruz Biotechnology, Dallas, TX, United States of America), p-mTOR (Santa Cruz Biotechnology, Dallas, TX, United States of America), glyceraldehyde 3-phosphate dehydrogenase (GAPDH) (Santa Cruz Biotechnology, Dallas, TX, United States of America) primary antibodies, and incubated with a secondary antibody at room temperature for 1 hour.

An enhanced chemiluminescence kit (Bio-Rad, Hercules, CA, United States of America) was used to visually detect proteins.

### RNA extraction and quantitative real-time polymerase chain reaction

The changes of gene expression in HSFs treated with SHI were observed by quantitative real-time polymerase chain reaction (qRT-PCR) and chloroquine (CQ, Sigma-Aldrich, St. Louis, MO, United States of America) as the control group. After 24 hours, total RNA was extracted from cells. A NanoDrop2000 spectrophotometer (Thermo Fisher, Wilmington, DE, United States of America) was used to measure RNA concentration, and RNA was reverse transcribed into cDNA, then qRT-PCR was carried out. The PCR thermocycling conditions were 95°C for 30 seconds, followed by under 95°C for 15 seconds and under 60°C for 30 seconds for 40 circles^18^.

To quantify the gene expressions of LC3 I/II, AMPK and ULK, the relative expression was analyzed by the 2^-ΔΔCt^ method and normalized to GAPDH. Each sample was analyzed at least three times. The qRT-PCR primers are shown in Table 1.

**Table 1:** Quantitative real-time polymerase chain reaction primers.

Genes	Sequences(5’–3’)	Annealing temperature (°C)
GAPDH	F: GGAAGCTTGTCATCAATGGAAATC	60
	R: TGATGACCCTTTTGGCTCCC	60
Beclin1	F:GGCACAATCAATAACTTCAGGC	60
	R: CCGTAAGGAACAAGTCGGTATCTC	60
MAP1LC3A	F:CAGACCGGCCTTTCAAGCA	60
	R: AAGGTTTCCTGGGAGGCGTA	60
AMPK	F:GGCAGAAGTATGTAGAGCAATCAAA	60
	R: GCAGTCCCTGATTTGGCTTCT	60
ULK1	F:CCTACACGCCATCTCCTCAAGT	60
	R: GACGTGCAAGTCAGACAGGTTG	60
P62	F:GGAAGGTGAAACACGGACACTT	60
	R: CTCTTCTCCTCTGTGCTGGAACT	60
mTOR	F:ATGCAGGAATAGCAAGAACTCG	60
	R: TCAGACCTCACAGCCACAGAAA	60

*Primers used in quantitative real-time polymerase chain reaction; F: forward; R: reverse. Source: Elaborated by the authors.

### Immunohistochemistry

Porcine full-thickness burn hypertrophic scar models were established as previously mentioned^11^. The porcine was randomly divided into two groups as a normal control injected with saline solution only and an SHI group injected with SHI only. The HS of porcine treated with saline solution and SHI were collected at zero, one, two, four and eight weeks and fixed by formalin for immunohistochemistry. After being dried, dewaxed, and rehydrated, the tissues were incubated with LC3 (Santa Cruz Biotechnology, Dallas, TX, United States of America) and P62 (Cell Signaling Technology, Beverly, MA, United States of America) overnight at 4°C.

### Statistical analysis

Statistical analysis was performed using Prism-GraphPad software 7.0. All data are expressed as mean ± standard error of the mean (SEM). Multiple comparisons were performed using ANOVA, and P < 0.05 was considered significant, while values of P < 0.01 were considered extremely significant.

## Results

### Inhibitory effects of Shikonin on hypertrophic scar-derived fibroblasts

The effect of SHI on cell growth ability and cell collagen expression was examined. The HSF cells had been incubated with 2, 1.75, 1.5 and 1.25 μM SHI for 24 hours. The SR method was used to measure the content of collagen in cells, and Alamar Blue (AB) was used to measure cell viability. As shown in Fig. 1c, the cellular morphological changes and collagen metabolism decreased with the increase of SHI concentration, compared with the control group. As shown in Fig. 1d, SHI significantly inhibited the growth of HSF cells in a dose-dependent manner, compared with the control group.

### Shikonin induced autophagy of hypertrophic scar-derived fibroblasts

Intracellular autophagosome aggregation of HSF with SHI treatment was observed by TEM in Fig. 1e. At 12 and 24 hours after treatment of cells with SHI, autophagosomes cumulatively increased compared to the control group, and ultrastructural changes were present. The amount of autophagy in HSF cells treated with SHI for 24 hours was higher than at 12 hours. At 12 hours, SHI-treated HSF cells appeared significantly edematous and rounded. The pseudopodia of the cell degenerated, the nucleus was large, and the nucleoli were marginally shifted. The mitochondria of the cell were abundant, swelling appeared, the intramembrane matrix became lighter, the individual endoplasmic reticulum stress closed in a ring to form autophagia, and autophagic lysosomes (ASS) were present in large quantities. The overall structure of cells in the control group was normal, the cell membrane was intact, and a small amount of ASS was present. At 24 hours, the HSF cells treated with SHI were significantly contracted and showed an apoptosis trend, and the organelles significantly increased, swelled, and aggregated. The mitochondria were abundant, slightly swollen, crest fractured and reduced, and ASS existed in large numbers. The overall cells of the control group were slightly edematous, and the number of ASS was small.

The SHI-induced autophagy related autophagosome changes were measured by an autophagy assay kit which provides a simple and direct procedure for measuring autophagy in HSFs. As indicated in Figs. 1f and 1g, autophagosome formation increased in SHI-treated HSFs (P < 0.01) compared with control groups after 24 hours. The result showed that autophagosomes increased in HSF stimulated with 1.75 μM SHI, which meant that SHI could induce autophagy in HSFs.

This study systematically investigated the underlying mechanisms of SHI in HSF using label-free quantitative proteomic analysis. The total of 3,554 proteins was quantified, and the functional analysis of the proteins was performed by GO enrichment. It was found that the differential proteins were significantly enriched on the autophagy-related biological process, as shown in Fig. 1h, and 18 differentially expressed proteins associated with autophagy were found, as shown in Table 2, which proved that SHI-induced HSF was closely related to the autophagy process.

**Table 2:** Autophagy-associated differential proteins.

Term	Category	Differential protein number	Protein_Gene			
Autophagy	Biological process	18	Accession	Gene name	Gene ID	Product
			P20073	ANXA7	310	Annexin A7
			Q9UN37	VPS4A	27183	Vacuolar protein sorting-associated protein 4A
			Q9H270	VPS11	55823	Vacuolar protein sorting-associated protein 11 homolog
			Q96JC1	VPS39	23339	Vam6/Vps39-like protein
			O75886	STAM2	10254	Signal transducing adapter molecule 2
			O95352	ATG7	10533	Ubiquitin-like modifier-activating enzyme ATG7
			Q6UWE0	LRSAM1	90678	E3 ubiquitin-protein ligase LRSAM1
			Q9NP79	VTA1	51534	Vacuolar protein sorting-associated protein VTA1 homolog
			Q9BRG1	VPS25	84313	Vacuolar protein-sorting-associated protein 25
			Q8IXJ6	SIRT2	22933	NAD-dependent protein deacetylase sirtuin-2
			Q96FZ7	CHMP6	79643	Charged multivesicular body protein 6
			Q8NEZ2	VPS37A	137492	Vacuolar protein sorting-associated protein 37A
			Q66K74	MAP1S	55201	Microtubule-associated protein 1S
			P15848	ARSB	411	Arylsulfatase B
			Q96AJ9	VTI1A	143187	Vesicle transport through interaction with t-SNAREs homolog 1A
			Q9HA65	TBC1D17	79735	TBC1 domain family member 17
			Q96EY5	MVB12A	93343	Multivesicular body subunit 12A
			Q96F24	NRBF2	29982	Nuclear receptor-binding factor 2

Source: Elaborated by the authors.

### Shikonin increases autophagy in hypertrophic scar-derived fibroblasts via the AMPK/mTOR signal pathway

**Figure 2:**
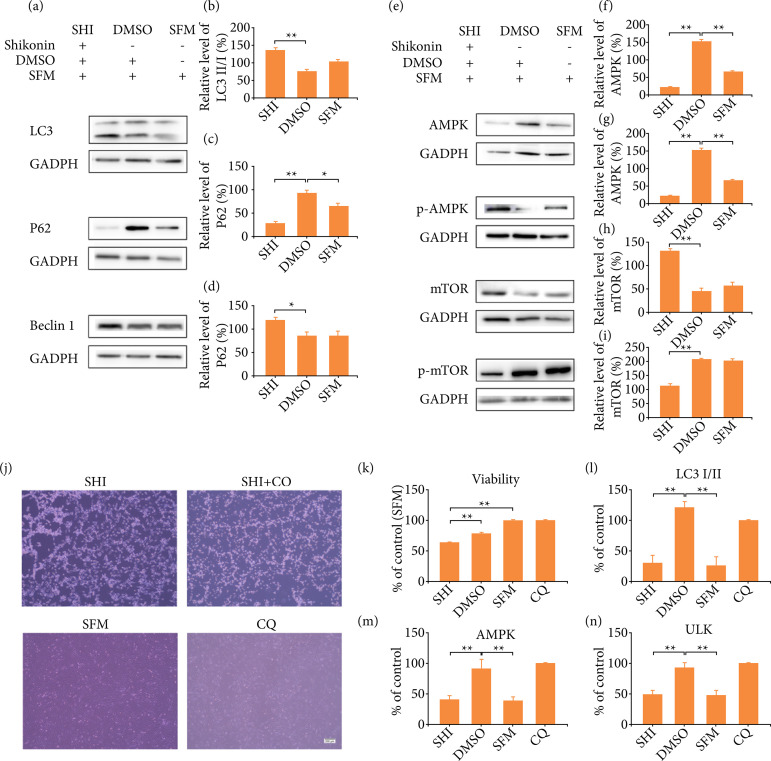
SHI increased autophagy in HSFs via the AMPK/mTOR signal pathway. **(a)** Western blotting was used to detect the changes of LC3II/I, P62 and Beclin1. GADPH as an internal control. **(b)** Quantification of the expression of LC3 II/I. **(c)** Quantification of the expression of P62. **(d)** Quantification of the expression of Beclin1. **(e)** Western blotting was used to detect the changes of AMPK, p-AMPK, mTOR and p-mTOR. GADPH as an internal control. **(f)** Quantification of the expression of AMPK. **(g)** Quantification of the expression of p-AMPK. **(h)** Quantification of the expression of mTOR. **(i)** Quantification of the expression of p-mTOR. **(j)** Representative images of HSFs treated with SHI, SHI + CQ, CQ and SFM and quantification of HSF viability. **(k)** Quantification of HSF viability. Relative protein expression levels of HSFs treated with SHI and CQ **(l)** LC3I/II, **(m)** AMPK, **(n)** ULK. Error bars represent the mean ± standard error of the mean

SHI: Shikonin; DMSO: methyl sulphoxide; SFM: serum free medium; HSF: hypertrophic scar-derived fibroblasts; LC3: Light Chain 3; CQ: cloroquine; n = 3; *significant difference with P < 0.01; **significant difference with P < 0.05. Source: Elaborated by the authors.

Western blot analysis was performed to study the expression levels of autophagy-associated proteins. As shown in Figs. 2a–2d, after 24 hours treatment of HSF cells with SHI, Western blotting results showed that the expression of LC3II/I and Beclin1 was significantly increased and P62 was significantly reduced compared to the control group, which suggested that SHI could induce autophagy in HSFs. It was also observed that SHI may regulate autophagy through the AMPK channel. As shown in Figs. 2e–2i, SHI upregulated the expression of p-AMPK and mTOR and downregulated the expression of AMPK and p-mTOR compared to the control group.

To further investigate the mechanism of SHI-induced autophagy, CQ was added to prevent autophagosome-lysosomal fusion, as shown in Figs. 2j and 2k. Combining SHI with the autophagy inhibitor CQ significantly increased cell activity compared to SHI alone, and SHI-promoting autophagy played an inhibitory role in the activity of HSFs. Changes in cell morphology after the addition of SHI were clearly seen. The CQ blocked the effect of autophagy on HSF viability in cells treated with SHI, and the cells that worked together with SHI and CQ were 13.6% ± 0.5% higher (P < 0.01) than cells treated with SHI alone. However, CQ did not change the morphology of cells after SHI treatment.

The qRT-PCR results showed that the expression of autophagy-related genes LC3I/II, AMPK and ULK in HSFs treated with SHI showed some inhibition in Figs. 2l–2n. The expression of LC3 I/II in SHI treatment groups was significantly lower than that of the SHI + CQ group (P < 0.01), and there was no significant expression of LC3I/II between SHI group and SFM group. Compared with SHI and SFM group, the accumulation of LC3-II in cells treated with CQ and SHI decreased, but the expression of AMPK and ULK in cells treated with SHI decreased significantly and increased significantly after treated with CQ.

### Protein expression in porcine full-thickness burn hypertrophic scar model

**Figure 3:**
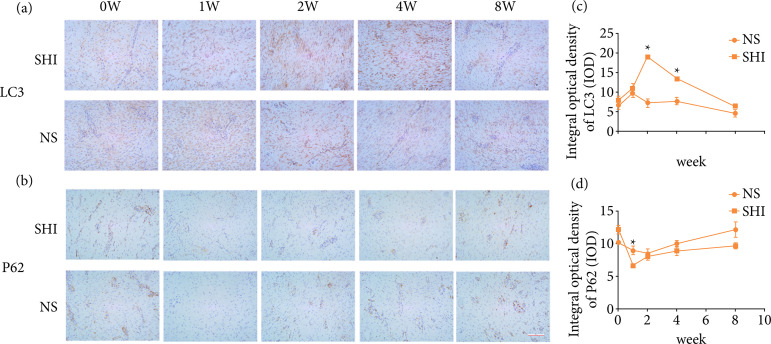
SHI affects the expression levels of LC3 and P62 proteins in hypertrophic scar of porcine during 0−8 weeks. **(a)** Immunohistochemical staining of LC3 staining at zero, one, two, four and eight weeks (20X). **(b)** Immunohistochemical staining of P62 staining at zero, one, two, four and eight (20X). **(c)** Quantification of LC3 staining. **(d)** Quantification of P62 staining.

SHI: Shikonin; NS: normal saline. Source: Elaborated by the authors.

The study established animal scar models to detect SHI-induced autophagy. Immunohistochemistry results showed in Figs. 3a–3d that the higher the antigen content, the higher the distribution density and the stronger the color of the positive result. Compared with the control group, the number of LC3-positive cells in the scar tissue of pigs in the SHI group was higher, while the expression of P62 was reduced. After the second week of treatment, LC3 expression was the highest in the SHI group (P < 0.05), and then slowly decreased over time, approaching the control group at the seventh week. The expression of P62 decreased one and two weeks after administration, after which it slowly returned to normal levels.

## Discussion

The formation of HS is mainly caused by excessive ECM deposition, as well as abnormal proliferation and migration of HSF^19^. Over-production of ECM is a common feature of many fibrotic diseases and may be induced by paracrine signals from activated lymphocytes and macrophages or autocrine factors secreted by fibroblasts^20^,^21^. In order to maintain tissue homeostasis, good balance between cell survival and cell death is necessary, which also be considered as the balance between autophagy and apoptosis^22^. Some articles suggest that scar hyperplasia and regression may be related to fibroblast autophagy and cell apoptosis^23^.

Autophagy is considered to be an essential regulator of fibrosis, tissue remodeling and ECM deposition^24^. It regulates the proliferation of fibroblasts through cytokines, degrades fibrin and reduces its deposition in the ECM. It has been shown that excessive autophagy stimulated fibroblast autophagy death, which could inhibit its proliferation and improve the formation of proliferative scars^25^,^26^. Other studies have found that the expression levels of LC3-II and Beclin1 in scar tissue were significantly reduced^13^. Autophagy and scar repair are very complex processes, and the mechanisms need to be explored from many angles.

Methods for detecting autophagy include fluorescence microscopy, Western blotting, flow cytometry and electron microscopy^27^. In this study, SHI was used to induce autophagy in HSF. Under electron microscopy, SHI was observed to increase mitochondrial vacuoles, organelle swelling, and abundant presence of ASS in cells. Other experimental results showed that SHI could induce autophagosome formation, and the presence of several-autophagy-related differentially expressed proteins, which was similar to hunger-induced autophagy, proving that SHI-induced HSF is closely related to the autophagy process.

The LC3, Beclin1 and P62 are important autophagy-related proteins^28^. The LC3 is now widely used to monitor autophagy, and, after autophagy occurs, LC3-I is converted to LC3-II, and the amount of LC3-II is clearly correlated with the number of autophagosomes^29^. The P62 is a ubiquitin-binding scaffold protein and can recruit phagophores by direct interacting with LC3-II^30^. It is low expressed when autophagy is induced and accumulates when autophagy is inhibited^31^. Beclin1, an autophagy specific protein, can regulate the formation of autophagosomes and induce and inhibit the autophagy pathway by interaction with several binding partners^32^. This study found that the upregulation and reduction of autophagy-related marker genes Beclin1 and LC3II/LC3I in HSFs indicated that autophagy could be activated after SHI treatment, so the activation of autophagy may be one of the mechanisms for repairing HS.

This study further examined the AMPK pathway of HSF cells at SHI-induced autophagy. Some reports suggested that the AMPK/mTOR signaling pathway was implicated in the regulation of autophagy^33^,^34^. Under stress conditions such as energy starvation, AMPK is activated as a sensor of energy molecules to regulate autophagy^35^ and is a positive regulator of autophagy that actively regulates autophagy by inhibiting mTOR activity^36^. The mTOR is a negative regulator of autophagy that inhibits autophagy by phosphorylating ULK1 to maintain it in an inactive state^37^.

This study found that SHI increased the expression of p-AMPK and decreased the expression of p-mTOR. It promoted AMPK phosphorylation and attenuated mTOR phosphorylation and can promote autophagy of HSFs. The mechanism of action may be related to inhibition of the activation of the AMPK/mTOR signaling pathway and affects the expression of downstream autophagy-related proteins, so it could be considered to be a drug that promotes autophagy.

The activity of the core proteins LC3II/LC3I, Beclin1, p-AMPK and mTOR was promoted, while P62, AMPK and p-mTOR activity was reduced, suggesting that SHI works by activating autophagy. In addition, CQ was used to further verify that SHI works through autophagy activation. The ULK is sensitive to metabolic state, and, under low energy or nutrient conditions, AMPK promotes autophagy by direct phosphorylation and activation of ULK1, while mTOR inhibits autophagy by inhibiting ULK1 activation^33^. Further experiments found that the autophagy flux inhibitor CQ could inhibit the conversion of LC3-I to LC3-II, causing the upregulation of AMPK and ULK activity, resulting in impaired autophagy flux.

To further determine whether SHI-promoted scar repair was associated with autophagy regulation in vivo, this study measured autophagy levels in porcine scar models over different time periods. Immunohistochemistry results showed that expression of LC3 II peaked after two weeks compared to the control group, suggesting that SHI promotes autophagy in porcine scar model. Overall, these results suggested that the AMPK/mTOR signaling pathway was involved in the SHI-promoting autophagy of HSFs, but whether SHI regulated other autophagy-related signaling pathways could not be completely ruled-out.

## Conclusion

This study demonstrated that SHI promoted autophagy of HSFs cells and may promote scar repair by inducing autophagy through the AMPK/mTOR signaling pathway. These findings supported the potential of SHI as a potential drug for the treatment of human HS and provided new targets for the early clinical treatment of HS. More research is needed to assess whether SHI is involved in other signaling pathways or the key molecular mechanisms behind autophagy and apoptosis regulation.

## Data Availability

Data during the current study will be available upon reasonable request.
